# A Review of Dry Eye Questionnaires: Measuring Patient-Reported Outcomes and Health-Related Quality of Life

**DOI:** 10.3390/diagnostics10080559

**Published:** 2020-08-05

**Authors:** Yuichi Okumura, Takenori Inomata, Nanami Iwata, Jaemyoung Sung, Keiichi Fujimoto, Kenta Fujio, Akie Midorikawa-Inomata, Maria Miura, Yasutsugu Akasaki, Akira Murakami

**Affiliations:** 1 Department of Ophthalmology, Juntendo University Graduate School of Medicine, Tokyo 113-0033, Japan; y-okumura@juntendo.ac.jp (Y.O.); n-iwata@juntendo.ac.jp (N.I.); jsung1@usf.edu (J.S.); k-fujimoto@juntendo.ac.jp (K.F.); k.fujio.zz@juntendo.ac.jp (K.F.); maria-k@juntendo.ac.jp (M.M.); y-akasaki@juntendo.ac.jp (Y.A.); amurak@juntendo.ac.jp (A.M.); 2 Department of Strategic Operating Room Management and Improvement, Juntendo University Graduate School of Medicine, Tokyo 113-0033, Japan; 3 Department of Digital Medicine, Juntendo University Graduate School of Medicine, Tokyo 113-0033, Japan; 4 Department of Ophthalmology, Juntendo University Faculty of Medicine, Tokyo 113-0033, Japan; 5 Department of Hospital Administration, Juntendo University Graduate School of Medicine, Tokyo 113-0033, Japan; ak-inomata@juntendo.ac.jp; 6 Morsani College of Medicine, University of South Florida, Tampa, FL 33612, USA

**Keywords:** dry eye disease, questionnaire, health-related quality of life, patient-reported outcome, OSDI, IDEEL, DEQS, UNC DEMS, CDERQOL, NEI VFQ-25

## Abstract

Dry eye disease (DED) is among the most common eye diseases and is becoming increasingly prevalent. Its symptoms cause a long-term decline in patients’ health-related quality of life (HRQL). Inconsistencies often occur between the clinical findings and the subjective symptoms of DED. Therefore, a holistic, balanced, and quantitative evaluation of the subjective symptoms and HRQL using patient-reported outcome questionnaires, in addition to clinical findings, is crucial for accurate DED assessment in patients. This paper reviewed the characteristics of current dry eye questionnaires, including their objectives, number of questions, inclusion of HRQL-related items, and whether they were properly evaluated for psychometric properties. Twenty-four questionnaires were identified; among them, the following six questionnaires that included items assessing HRQL and were properly evaluated for psychometric properties are recommended: the Ocular Surface Disease Index, Impact of Dry Eye in Everyday Life, Dry Eye-Related Quality-of-life Score, University of North Carolina Dry Eye Management Scale, Chinese version of Dry Eye-Related Quality of Life, and 25-Item National Eye Institute Visual Function Questionnaire. Dry eye questionnaires have different objectives and are available in different languages. Therefore, medical practitioners should confirm the characteristics of applicable questionnaires before selecting the most appropriate ones.

## 1. Introduction

Dry eye disease (DED) is among the most common eye diseases worldwide [1]; its prevalence is expected to increase even further given the increasing use of visual display terminals, increasing aging population, and highly stressful social environments [2,3,4,5]. DED causes ocular surface damage, eye discomfort, and visual impairment; moreover, it reduces the health-related quality of life (HRQL) and work productivity [6,7,8,9,10,11,12]. DED diagnosis is based on the quantification of subjective symptoms and dry eye examinations, including tear film breakup time (TFBUT), ocular surface staining, tear secretion volume measurement using Schirmer’s test, and tear osmolarity [12,13]. According to The TFOS DEWS II Diagnostic Methodology report [13], the diagnosis of dry eye symptoms should be initially performed by excluding non-DED possibilities from the differential diagnosis through triage questions, followed by an assessment of the patient’s risk factors for DED. Notably, at least one positive finding among the markers for tear film homeostasis loss should constitute DED diagnosis [13]. However, inconsistencies often occur among subjective symptoms, HRQL, and the examination-based clinical findings of patients with DED [12], since many of them lack subjective symptoms or experience a lower HRQL than that suggested by their clinical findings [14,15,16,17]. A questionnaire may lack sensitivity, particularly in cases with neurotrophic alterations due to or accompanied by DED, which could result in a decreased corneal sensation and hence less symptomology [18,19]. Therefore, a holistic, balanced consideration of the quantitative measurements of subjective symptoms and HRQL (via patient-reported outcome questionnaires), in addition to clinical findings, is required for the accurate assessment and treatment of DED. The TFOS DEWS II Diagnostic Methodology report indicates that the quantification of subjective symptoms is one of the primary methods of dry eye diagnosis [13]. Furthermore, according to the dry eye diagnostic criteria proposed by the Asia Dry Eye Society in 2016, DED diagnosis should be based on the presence of positive findings in two distinct factors, i.e., subjective symptoms and reduced TFBUT. Consequently, it has become increasingly important to quantify subjective dry eye symptoms [10,13].

In addition to the medical indicators based on the physiological tests, there has been a recent emphasis on patient-reported outcomes (PROs) in therapeutic efficacy evaluation [20]. To this effect, there are currently proactive efforts in the United States toward implementing corresponding methodologies under the policy guidance of the Food and Drug Administration (FDA) [21] and the Patient-Centered Outcomes Research Institute established through the Affordable Care Act [22,23]. According to the PRO guidance of the FDA, questionnaires should document the development process, psychometric characteristics, and responsiveness of the device to ensure its relevance and suitability for the target population, as well as its intended use. PROs are intra-treatment indicators based on patients’ subjective evaluations of relevant aspects, including disease symptoms, satisfaction with treatment, and HRQL, which are obtained through interviews and questionnaires. PROs allow disease evaluation by medical personnel and patients to complement the unilateral evaluation by medical personnel solely based on examination findings. Appropriate PRO measurement methods are required to ensure validity and reliability while accounting for the subjective index characteristics [20]. To achieve this, questionnaires should be developed through scientifically valid processes, including psychometric property evaluation [14,24]. Psychometric properties refer to the validity and reliability of clinical tools, including paper-based clinical questionnaires. Measurement validity is divided into numerous categories, including content validity, concurrent validity, construct validity, and criterion-related validity [25]. Furthermore, measurement reliability comprises subcategories, including internal consistency and test–retest reliability. According to the PRO guidance document released by the FDA, a clinical questionnaire should demonstrate satisfactory levels of psychometric properties before being used in practice.

Future improvements in personalized medicine for dry eye treatment are expected [16,17,26,27]. In personalized medicine, rather than employing standardized treatment, the treatment methods are based on the severity and extent of an individual’s condition and subjective symptoms [17]. Therefore, the accurate evaluation of subjective symptoms will play an increasingly significant role in medical care.

Currently, there are various questionnaires used in clinical practice regarding subjective dry eye symptoms. These questionnaires greatly differ with respect to when they were created, languages used, number of questions, and the content of the questions. Moreover, the psychometric properties of some questionnaires remain unexamined, while other questionnaires cannot accurately determine PROs. Therefore, during dry eye treatment, medical practitioners should consider the characteristics of each relevant questionnaire for appropriate selection. This paper reviews the various characteristics of numerous dry eye questionnaires, including their objectives, number of questions, inclusion of questions regarding HRQL, and the evaluation of psychometric properties.

## 2. Literature Review

This literature review of dry eye questionnaires was performed at the Department of Ophthalmology, Juntendo University Faculty of Medicine between December 2019 and January 2020. The terms “dry eye” and “questionnaire” were used for the initial broad-term search, with the inclusion of additional search terms as deemed necessary, eventually identifying 24 questionnaires. Notably, a significant number of the questionnaires were developed before the release of the FDA’s PRO guidance document. Therefore, this report on the psychometric properties (validity and reliability) based on the PRO guidance is relevant for future application by practitioners. Specifically, this study reviewed whether each questionnaire had clinical relevance, known group validity, concurrent validity, internal consistency, and reproducibility using Hunsley and Mash’s criteria [28]. Furthermore, based on previous literature, this study reports whether the validity of uni-dimensional evaluation was confirmed using Rasch analysis. The 24 questionnaires were categorized into six groups based on whether they included HRQL questions, as well as their psychometric properties as follows: good results indicated by sufficient research, insufficient or poor results, or no conducted survey.

Table 1 summarizes the results classified into six categories. Table 2 summarizes the questionnaire characteristics, including the year of development, number of questions, and validation language. The following text presents detailed information regarding each questionnaire.

### 2.1. Questionnaires with Questions on HRQL and Good Psychometric Properties

#### 2.1.1. Ocular Surface Disease Index (OSDI)

The OSDI questionnaire is a 12-item instrument that was developed in 1997 [29]. This questionnaire, which has good psychometric properties, was created to assess subjective dry eye symptoms and the effects of DED on the vision-related activities of daily living within the previous week [24,30]. This questionnaire has three subscales: ocular symptoms (three questions), vision-related functions (six questions), and environmental triggers (three questions), with each question being answered on a five-point scale ranging from “None of the time” (no points) to “All of the time” (four points) (N/A is selected when the question is not applicable). The OSDI total score ranges from 0 to 100 points and is obtained by multiplying the total score of all the questions by 25 and dividing the result by the number of valid answers. Using this method, a maximum score of 100 points can be obtained per subscale. The total score is positively correlated with DED severity and the impact on activities of daily living. The OSDI total score can be used to classify the respondent’s dry eye symptoms as normal (0–12 points), mild (13–22 points), moderate (23–32 points), or severe (33–100 points) [30,31,32]. This questionnaire was originally developed in English [29] with subsequent translations and validations in Portuguese [33], Spanish [34], Farsi [35], Bahasa [36], Chinese [37], Filipino [38], and Japanese [39]. However, caution should be applied when using it, given that the cutoff values for DED differ across languages, with a threshold of 27.2 and 36.3 points in the Chinese and Japanese versions, respectively [37,39,40]. The OSDI has good concurrent validity, internal consistency (Cronbach’s alpha = 0.78–0.92), and test–retest reliability (intraclass correlation coefficient (ICC) = 0.70–0.82) [24,30]. Previous studies have reported good correlations between the OSDI and other questionnaires, including the McMonnies questionnaire (Spearman correlation coefficient, r = 0.52–0.67, *p* < 0.001), Dry Eye Questionnaire (DEQ) (Spearman correlation coefficient, r = 0.76, *p* < 0.05), 25-Item National Eye Institute Visual Function Questionnaire (NEI VFQ-25) (Spearman correlation coefficient, r = −0.55–−0.77, *p* < 0.001), Ocular Comfort Index (OCI) (Spearman correlation coefficient, r = 0.68–0.78, *p* < 0.001), and Subjective Evaluation of Symptom of Dryness (SESoD) (Spearman correlation coefficient, r = 0.75, *p* < 0.05) [41,42]. The clinical validity depends on the study population and design; therefore, the findings vary across studies. However, the OSDI has either no or a very slight correlation with clinical parameters [41,43] and can be used for DED diagnosis and HRQL evaluation in patients with DED with comorbidities, including glaucoma and bronchial asthma [44,45,46,47]. Recently, Inomata et al. validated a smartphone application-based OSDI questionnaire, which was derived from the paper-based questionnaire [16]. The OSDI is limited in that it does not address all dry eye symptoms, including foreign body sensation [48]. A recent study that employed Rasch analysis reported that dividing the response items into four categories by combining “half of the time” and “most of the time” could result in higher thresholds and improved intervals in each category [49].

#### 2.1.2. Impact of Dry Eye in Everyday Life (IDEEL)

The IDEEL questionnaire is a 57-item instrument developed in 2003 [50]. This questionnaire has good psychometric properties and was created to assess subjective dry eye symptoms, the vision-related functions of daily living, and satisfaction with DED treatment within the previous two weeks [24,48,51]. It comprises three modules: dry eye impact on daily life, dry eye treatment satisfaction, and dry eye symptom bother. Except for the questions with yes or no responses, the remaining questions are answered using a 4- or 5-point Likert scale, with the score of each module ranging from 0 to 100 points. There is a positive correlation of the score for dry eye impact on daily life and treatment satisfaction with the patient’s HRQL and satisfaction level with treatment. Moreover, the dry eye symptom score is positively correlated with the DED-caused inconvenience level [48]. This questionnaire lacks cutoff values for DED. However, the mean score of the dry eye symptom bother module for patients with mild, moderate, and severe DED was reported to be 40.0 (±7.5), 50.6 (±11.0), and 64.3 (±8.0) points, respectively [52]. Moreover, the Minimal Clinically Important Difference (MCID) [53] in this module was reported to be 12 points [52]. This questionnaire was originally developed in English, with the subsequent development of the Chinese version of the Dry Eye-Related Quality-of-Life (CDERQOL) questionnaire [54]. The IDEEL questionnaire involves tests based on the FDA guidance on PRO instrument development and has good validity and reliability, including internal consistency (Cronbach’s alpha = 0.70–0.97) and test–retest reliability (ICC = 0.70–0.88) [24,48,51]. It is weakly correlated with other dry eye questionnaires, including the DEQ (Pearson coefficient ranges: –0.05–0.83) and general quality-of-life (QoL) questionnaires (Short Form-36 and EuroQoL-5D) [51]. The limitations of this questionnaire include the need for users to purchase it and the time required—approximately 30 min—to complete it given the large number of questions.

#### 2.1.3. Dry Eye-Related Quality-Of-Life Score (DEQS)

The DEQS questionnaire is a 15-item instrument developed in 2013 for assessing subjective dry eye symptoms and their effects on activities of daily living within the previous week [55]. It has a clear development process based on PRO guidance [21] and good psychometric properties [55]. The DEQS questionnaire comprises six questions on eye symptoms and nine on the dry eye effects on activities of daily living. Each question has a column A and B for the frequency and severity, respectively. Responses to the frequency portion in column A are based on a five-point scale ranging from “None of the time” (no points) to “All of the time” (four points). Here, a score of 1 to 4 points prompts the respondent to proceed to column B to answer the questions regarding severity on a four-point scale. A QoL score ranging from 0 to 100 points is calculated by multiplying the total points in column B by 25 and dividing the result by the number of valid responses. The QoL score is positively correlated with the severity of subjective dry eye symptoms and dry eye effects on daily life. The cutoff value for DED is 15 points [56]. This questionnaire was developed in Japanese and has not been validated in other languages. The DEQS has a clearly defined development process and was created according to the guidance for PRO device development published by the FDA. A psychometric analysis confirmed this questionnaire’s validity and reliability, including its internal consistency (Cronbach’s alpha = 0.83–0.93) and test–retest reliability (ICC = 0.81 to 0.93) [55]. The DEQS score is significantly correlated with the NEI VFQ-25 (Pearson coefficient ranges: −0.20–0.77) [55]. The limitation of this questionnaire is that it is not validated in languages other than Japanese, which limits its usage in other nations.

#### 2.1.4. University of North Carolina Dry Eye Management Scale (UNC DEMS)

The UNC DEMS is a single-item questionnaire that was developed in 2014 [57] to assess subjective dry eye symptoms and their effects on activities of daily living within the previous week. It has a clear development process based on the PRO guidance [19] and good psychometric properties. Questions regarding subjective DED symptoms and their effects on daily life are answered on a 1–10 scale. The score is positively correlated with symptom severity and its impact on daily life. It lacks a designated cutoff value; however, the reported mean score for patients with and without DED is 5.73 (±2.15) and 1.85 (±1.72) points, respectively. A recent study reported that the MCID was 1 point [58]. The UNC DEMS was developed in English and is yet to be validated in other languages. The UNC DEMS was developed in accordance with the Patient-Reported Outcome Measurement Information System, which was established by the National Institutes of Health following the guidance for PRO device development published by the FDA [59]. This questionnaire has been reported to have good validity and reliability, including test–retest reliability (ICC = 0.84–0.95), has been explored and yielded good results. The questionnaire is strongly correlated with the OSDI (Pearson coefficient ranges: 0.69–0.87, *p* < 0.001) and moderately correlated with clinical findings, including TFBUT [57].

#### 2.1.5. Chinese Version of Dry Eye-Related Quality of Life (CDERQOL)

The CDERQOL questionnaire is a 45-question questionnaire developed in 2017 [54] to assess the subjective dry eye symptoms and their effects on vision-related activities of daily living within the previous two weeks. Its development process based on the PRO guidance [19] is clear and has good psychometric properties. The CDERQOL was created in Chinese based on the IDEEL questionnaire [48] and broadly comprises three domains: dry eye symptom bother (12 questions), dry eye impact on daily life (24 questions), and satisfaction with treatment (nine questions). The dry eye impact on daily life domain is further divided into three domains: impact on daily activities (seven questions), emotional impact (10 questions), and impact on work (seven questions). Each question is answered on a five-point Likert scale that ranges from “completely disagree” (one point) to “completely agree” (five points). The score is positively correlated with symptom severity and its impact on daily life. There is no designated cutoff value; however, the scores of all domains, except for those for the domain of satisfaction with treatment, significantly differed according to the DED severity (in the dry eye symptom bother domain, the mean scores for patients with mild, moderate, and severe dry eye were 32.7 (±8.4), 35.3 (±9.8), and 44.1 (±9.0) points, respectively). Its development process based on the PRO guidance [19] is clear and has good psychometric properties including construct validity and internal consistency (Cronbach’s alpha = 0.72 to 0.91) [54]. Its limitations include the small sample size used for the psychometric analysis and the lack of validation in languages other than Chinese [60].

#### 2.1.6. The 25-Item National Eye Institute Visual Function Questionnaire (NEI VFQ-25)

The NEI VFQ-25 questionnaire is a 25-item questionnaire developed in 2001 [60] for assessing the HRQL for vision at the time of responding to the questionnaire and without any set timeframe. It is not specifically designed for DED; however, it has verified psychometric properties [60]. It is the short version of the National Eye Institute Visual Function questionnaire, which comprises 51 questions [61]. It has an additional 14 questions for more sensitive measurements. Each question is answered on several scales. Subsequently, each item is converted to a 0 (lowest possible score) to 100 (highest possible score) scale. Its score is positively correlated with the HRQL and it lacks a designated cutoff value for DED. However, in the pain subscale, the mean scores for patients with moderate and mild DED were 60.8 (±14.1) and 71.8 (±19.2) points, which showed that patients with moderate DED had a significantly lower score [62]. This questionnaire has been validated for use in more than 50 languages [63,64,65,66,67,68,69,70,71]. The NEI VFQ-25 has good concurrent validity, internal consistency (Cronbach’s alpha = 0.71 to 0.85), and test–retest reliability (ICC = 0.57 to 0.88) [60,62]. Compared with the OSDI, it has moderate concurrent validity (Spearman correlation coefficient, r = 0.61) [42]. It is not significantly correlated with the overall McMonnies questionnaire; however, it has a weak correlation with the pain subscale (Spearman correlation coefficient, r = 0.28) [72]. Its limitations include the lack of specificity for DED and the evaluation of psychometric properties in patients with DED.

### 2.2. Questionnaires with Questions on HRQL, Yet Inadequate Examination or Poor Results for Psychometric Properties

#### 2.2.1. Dry Eye Questionnaire (DEQ)

The DEQ questionnaire is a 21-item instrument that was developed in 2001 [73] for DED diagnosis and severity assessment; however, its psychometric properties are inadequately examined. The DEQ involves a seven-day timeframe with each question being answered using a Likert scale. The questionnaire includes questions on HRQL; however, it lacks a designated cutoff value for DED. The questionnaire remains to be validated in other languages. The uni-dimensionality of each question has been assessed using Rasch analysis; moreover, it has concurrent validity with the OSDI and McMonnies questionnaires (Spearman correlation coefficients, r = 0.76 and 0.66, respectively). However, the examinations of its reliability were missing [41].

#### 2.2.2. Ocular Surface Disease (OSD)

The OSD is an 80-item questionnaire that was first reported in 2002 [74]. It was created for the diagnosis of ocular surface disease and comprehensive HRQL evaluation. Among its sections, only the psychometric properties of the QoL section (OSD-QoL) have been examined with good results. This questionnaire has an undefined timeframe and comprises four sections: symptom, medical, satisfaction with treatment, and OSD-QoL. It was originally developed in French; subsequently, in 2015, an English version of the OSD-QoL section that comprised 28 questions was created to allow the characterization of the effects of ocular surface disease on the HRQL of patients [75]. However, other sections have not been validated in languages other than French. The OSD-QoL section is further divided into seven dimensions: “Daily activities” (five questions), “Difficulties with work and handicap” (five questions), “Giving up make-up” (one question), “Acknowledgement of the disease condition” (two questions), “Acceptance of the disease” (one question), “Fear for the future” (five questions), and “Emotional well-being” (nine questions). Each dimension was evaluated based on a total possible score of 100 points and the score is positively correlated with the HRQL. The OSD-QoL is unspecific for DED and lacks a designated cutoff value for it. Among the four sections, only the OSD-QoL section has been examined for psychometric properties with good validity and reliability, including construct validity and internal consistency (Cronbach’s alpha = 0.81–0.92; the Cronbach’s alpha value for the “acknowledgement of the disease” was 0.40). Consequently, an overall examination of the psychometric properties of the OSD questionnaire is missing [75].

#### 2.2.3. Texas Eye Research and Technology Center Dry Eye Questionnaire (TERTC-DEQ)

The TERTC-DEQ questionnaire is a 28-item instrument that was developed in 2005 [73] to identify the patients with moderate DED and evaluate the severity of subjective dry eye symptoms. However, its psychometric properties have been inadequately examined [73,76] and it has an undefined timeframe. This questionnaire is derived from the DEQ and includes two questions on HRQL, which are too few. Each question is answered using a 0–4 (or 0–1) Likert scale after which the total score is obtained. The score is positively correlated with the severity of subjective dry eye symptoms. The cutoff value for moderate DED is 32.3 points. The questionnaire was developed in English and remains to be validated in other languages. Despite it showing internal consistency (Cronbach’s alpha = 0.95) and a correlation with the McMonnies questionnaire (Spearman correlation coefficient, r = 0.51, *p* = 0.003), the Bland–Altman test showed significant variability in its test–retest reliability [76]. Therefore, its reliability remains inadequately examined.

### 2.3. Questionnaires with Questions on QoL, Yet Missing Examination of Psychometric Properties

#### 2.3.1. Contact Lens Dry Eye Questionnaire (CLDEQ)

The CLDEQ questionnaire is a 36-item instrument that was developed in 2001 [77]. It was derived from the DEQ to specifically examine the distribution of dry eye symptoms among contact lens wearers. However, its psychometric properties are missing. The questionnaire includes questions regarding the subjective symptoms among contact lens wearers with the remaining question being similar to those in the DEQ [73]. Like the DEQ, the CLDEQ considers a 7-day timeframe and contains questions on HRQL; however, it lacks a designated cutoff value for DED and has not been validated in other languages.

#### 2.3.2. The 11-Question Dry Eye Syndrome Questionnaire

The 11-question dry eye syndrome questionnaire was developed in 2007 [12] to evaluate the effects of DED on the vision-related activities of daily life within the previous week. However, its psychometric properties have not been evaluated. Each global question is answered using a 0–10 scale. This questionnaire assesses the effects of DED on activities of daily living (reading, professional work, TV watching, and driving) with the following answers: all of the time, most of the time, half of the time, some of the time, none of the time, or not applicable. It does not have a designated cutoff value. The questionnaire was developed in English and is yet to be validated in other languages.

### 2.4. Questionnaires with No Questions on HRQL, Yet Good Psychometric Properties

#### Ocular Comfort Index (OCI)

The OCI questionnaire is a 12-item questionnaire that was developed in 2007 [78] to evaluate eye symptom frequency and severity within the previous week. Moreover, it has good psychometric properties but lacks questions on HRQL. Questions on eye symptom frequency are answered using a seven-point scale that ranges from “Never” (0 points) to “Always” (6 points). Similarly, symptom severity is also answered on a seven-point scale ranging from “Never had it” (0 points) to “Severe” (6 points). A score from 0 to 100 is obtained by entering the results into the OCI Calculator [78]. The score is positively correlated with eye symptom severity. The questionnaire lacks a designated DED cutoff value. It was developed in English and has been validated in Chinese as the Chinese version of the Ocular Comfort Index (OCI-C) [79]. Its development process has been documented using Rasch analysis and it has been psychometrically validated, including its construct validity and test–retest reliability (ICC = 0.81 to 0.91) [78]. It has a moderate positive and negative correlation with the OSDI (Spearman correlation coefficient, r = 0.68–0.78, *p* < 0.001) and TFBUT (Spearman correlation coefficient, r = −0.23–−0.56, *p* < 0.0001), respectively [78]. Rasch analysis has shown that the OCI-C is comparable to the OCI as a diagnostic tool [79].

### 2.5. Questionnaires with No Questions on HRQL and Inadequate Examination or Poor Results for Psychometric Properties

#### 2.5.1. McMonnies Questionnaire

The McMonnies questionnaire is a 12-item questionnaire that was developed in 1986 [80] for DED screening and evaluating DED risk factors. Its psychometric properties have been insufficiently examined; furthermore, it lacks questions on HRQL. The questionnaire does not clearly define a target timeframe. Each question is individually scored and the McMonnies Index (perfect score = 45) is calculated by summing the scores. The severity of dry eye symptoms is positively correlated with the McMonnies Index. The cutoff value of the McMonnies Index for DED diagnosis is 14.5 points [72,81]. This questionnaire has been validated in Chinese, with its McMonnies Index cutoff value being 15 points [37]. The McMonnies questionnaire has a weak positive correlation with the NEI VFQ-25 pain subscale (Spearman correlation coefficient, r = 0.28) [72], and each question has good uni-dimensionality [41]. However, it has poor internal consistency (Cronbach’s alpha = 0.43) [72], and its validity and reliability have not been adequately examined. A study using Rasch analysis concluded that the questionnaire is unsuitable for DED severity evaluation [82].

#### 2.5.2. Schein Questionnaire

The Schein questionnaire is a six-item questionnaire that was developed in 1997 [83] to evaluate DED prevalence among the elderly. Its psychometric properties have been inadequately examined, and it lacks questions on HRQL. The questionnaire lacks a clearly defined target timeframe. Respondents are asked to report the frequency of subjective symptoms (rarely, sometimes, often, or all of the time). Since the answers are not scored, there is no cutoff value for DED. The questionnaire was developed in English and has not been validated in other languages. It has a moderate internal consistency (Cronbach’s alpha = 0.61); however, its validity was missing [83,84,85].

#### 2.5.3. Women’s Health Study Questionnaire

The Women’s Health Study questionnaire is a three-item questionnaire that was developed in 2001 [86] to evaluate the DED prevalence in women in the USA [87]. Its psychometric properties have not been adequately examined and it lacks questions on HRQL. The questionnaire lacks a clear definition of a target timeframe and comprises two questions on the history of dry eye diagnosis and dry eye symptom frequency. Questions regarding frequency are answered using the following terms: constantly, often, sometimes, or never. Since the answers are not scored, there is no cutoff value. The questionnaire was developed in English and has not been validated in other languages. It has good test–retest reliability (ICC = 0.75) [88]. However, other validity examinations were missing.

#### 2.5.4. Standard Patient Evaluation of Eye Dryness Questionnaire (SPEED)

The SPEED is a 20-item questionnaire that was developed in 2005 [89] to assess the severity and changes in the subjective symptoms experienced over time by patients with DED. Its psychometric properties have been inadequately examined; moreover, it lacks questions on HRQL [89,90]. Each question has a different timeframe. The questionnaire is divided into three sections associated with the presence or absence, frequency, and severity of symptoms. Currently, there are three different timeframes (e.g., now, the last 72 h, and the last 3 months) available for questions assessing the presence or absence of symptoms. Questions on symptom frequency and severity are answered on a 0–3 and 0–4 Likert scale, respectively. It does not have a designated cutoff value. The questionnaire was developed in English and is yet to be validated in other languages. The questionnaire has been evaluated using Rasch analysis. In 2013, a comparative study was conducted to compare this questionnaire with the four main questionnaires on DED (OSDI, DEQ, McMonnies questionnaire, and SESoD). It was found to have uni-dimensionality and was comparable in identifying DED [91]; however, its other validity and reliability attributes have not been examined. There is a good correlation between the SPEED and the meibomian gland and meibomian glands yielding liquid secretion scores, which are the scales for evaluating meibomian gland dysfunction (MGD), one of the main DED causes. Therefore, this questionnaire is useful for MGD evaluation [91].

#### 2.5.5. Symptom Assessment in Dry Eye (SANDE)

The SANDE questionnaire is a two-item instrument that was developed in 2007 [92] to quantify dry eye symptom frequency and severity within the previous two months. Its psychometric properties have not been adequately examined and it lacks questions on HRQL. The questionnaire has two versions—1 and 2—based on a visual analog scale (VAS) using the 100 mm horizontal VAS technique. Respondents are asked to answer two questions on the frequency and severity of dry eye symptoms. In version 1, the responses to the questions range from (far left to far right) “Rarely” to “All the time” for frequency, or “Very mild” to “Very severe” for severity. The distance of each response from the far left is measured in mm and the score is obtained by multiplying and squaring the values. Version 2 is recommended for comparison with previous results. Here, the last visit scale is set as the central anchor at 5 mm from the far left. Responses to questions on frequency range from (far left to far right) “Much less frequent” to “Much more frequent”; those for the severity range from “Much less severe” to “Much less severe”. In version 2, the right and left central anchors are positive and negative, respectively. The distance (in mm) from the central anchor is used as the score. It does not have a designated cutoff value. The questionnaire was developed in English and is yet to be validated in other languages. The baseline SANDE and OSDI scores are significantly correlated at the baseline visit (Spearman correlation coefficient, r = 0.64, *p* < 0.0001); moreover, changes in the SANDE and OSDI score from baseline to follow-up visits were significantly correlated (Spearman correlation coefficient, r = 0.47, *p* < 0.0001) [93]. However, examinations of its reliability were missing.

#### 2.5.6. Dry Eye Questionnaire-5 (DEQ-5)

The DEQ-5 is a five-item questionnaire that was developed in 2009 [43]. It is the short version of the DEQ [73] and was created to evaluate dry eye symptom severity within the previous month. Its psychometric properties have not been adequately examined, and it lacks questions on HRQL. Each question is answered on a 0–4/0–5 Likert scale, where the total score is obtained and evaluated based on a 0–22 scale. It has designated cutoff values where scores of >6 and ≥12 points are suggestive of DED and Sjogren’s syndrome, respectively. The questionnaire was developed in English and has been subsequently validated in Spanish. Similarly, a score of ≥ 6 in the Spanish version is suggestive of DED [94]. The DEQ-5 questionnaire is correlated with the questionnaire of the Self-Assessment of Severity (Spearman correlation coefficient, r = 0.35 to 0.48, *p* < 0.01). However, other validity and reliability factors have not been examined.

### 2.6. Questionnaires without Questions on HRQL and No Examination of Psychometric Properties

#### 2.6.1. Canadian Dry Eye Epidemiology Study (CANDEES)

The CANDEES is a 13-item questionnaire that was developed in 1997 [95] to investigate the DED prevalence in Canada. Its psychometric properties have not been evaluated and it lacks questions on HRQL. It lacks a target timeframe and its questions employ a mark sheet format to assess the respondent’s history of systemic diseases and the presence of dry eye symptoms. Since its answers are not scored, it lacks a cutoff value. The questionnaire was developed in English and is yet to be validated in other languages.

#### 2.6.2. Dry Eye Screening Questionnaire for Dry Eye Epidemiology Projects (DEEP)

The DEEP questionnaire is a 19-item questionnaire that was developed in 1998 [96] to screen patients with DED in epidemiologic studies. Its psychometric properties have not been evaluated and it lacks questions on HRQL. The questionnaire lacks a clearly defined target timeframe. Questions on symptom frequency are answered using a four-point scale (Constantly, Often, Sometimes, Never) while yes/no questions are answered using a three-point scale (Yes, No, Don’t know). Moreover, all questions are scored using an equivalent scoring system (Constantly = 6; Yes = 6; Often = 4; Don’t know = 3; Sometimes = 2; No = 0; Never = 0) with the total score ranging from 0 to 114 points. A previous study that performed a receiver operating characteristic analysis using 14 out of 19 questions to investigate sensitivity and specificity did not report clear cutoff values [96]. The questionnaire was developed in English and is yet to be validated in other languages.

#### 2.6.3. McCarty Symptom Questionnaire

The McCarty symptom questionnaire is a six-item questionnaire that was developed in 1998 [97] to investigate the prevalence of DED in Australia [97,98]. Its psychometric properties have not been evaluated, and it lacks questions on HRQL. It lacks a target timeframe and the six questions on dry eye symptoms are answered using a scale ranging from “Absent” (grade 0) to “Severe” (grade 3). It lacks a designated cutoff value. The questionnaire was developed in English and is yet to be validated in other languages.

#### 2.6.4. Sicca Questionnaire (Sicca Symptom Inventory, SSI)

The SSI questionnaire is a 42-item questionnaire that was developed in 2003 [99] to evaluate the severity of dryness symptoms (eyes, mouth, vagina, skin) in primary Sjogren’s and Sicca syndromes. Its psychometric properties have not been evaluated and it lacks questions on HRQL. The target timeframe of the questionnaire is the previous two weeks. The long and short forms of this questionnaire comprise 42 and 19 questions, respectively [99,100]. The questionnaire comprises 10 domains: three on the eyes, five on the mouth, one on the vagina, and one on the whole body and joints. Answers to each question are scored on a 0–4 Likert scale that ranges from “Never” (no points) to “All the time” (four points). Higher scores indicate more severe symptoms. It lacks a designated cutoff value. The questionnaire was developed in English with the short form being subsequently validated in Portuguese [101]. All scores for the three eye domains are correlated with the OSDI and weakly correlated with clinical findings [99,102].

#### 2.6.5. Single Item Score Dry Eye Questionnaire (SIDEQ)

The SIDEQ is a single-item questionnaire that was developed in 2003 [103] to evaluate whether the respondent had dry eye symptoms at the time of answering the questionnaire. Its psychometric properties have not been evaluated and it lacks questions on HRQL. Its questions on DED-induced eye discomfort are answered using a five-point scale ranging from “None” (no points) to “Severe” (four points). Higher scores indicate more severe symptoms. It lacks a designated cutoff value. The questionnaire was developed in English and has not been validated in other languages. The SIDEQ is strongly correlated with the OSDI and clinical findings [24].

#### 2.6.6. Contact Lens Dry Eye Questionnaire-8 (CLDEQ-8)

The CLDEQ-8 is an eight-item questionnaire that was developed in 2009 [104]. It is the short version of the CLDEQ [77] and was created to evaluate the severity of dry eye symptoms in soft contact lens wearers within the past 2 weeks [105]. Its psychometric properties have not been evaluated and it lacks questions on HRQL. Each question is answered using a 0–4, 0–5, or 1–6 Likert scale where the total score is obtained and evaluated based on a 0–37 scale. The cutoff value for DED is set to ≥ 12 points [106]. The questionnaire was developed in English and has been validated in Japanese as the Japanese CLDEQ-8 (J-CLDEQ-8); the cutoff value for the J-CLDEQ-8 is 11 points [107]. The CLDEQ-8 can evaluate changes in subjective symptoms when changing contact lens type.

## 3. Discussion

There are often inconsistencies between the clinical findings and subjective symptoms of DED [12,14,15,16,17]; moreover, DED adversely affects patients’ HRQL [6,7,8,9,10,11,12]. Therefore, there is a need to accurately determine the subjective DED symptoms and their impact on a patient’s daily life through reliable and valid questionnaires created in accordance with the PRO guidance. There are many DED-related questionnaires; however, most only assess subjective symptoms, with few having been adequately evaluated for psychometric properties. This review identified that six questionnaires, namely, the OSDI, IDEEL, DEQS, UNC DEMS, CDERQOL, and NEI VFQ-25, all of which contained questions regarding HRQL and were fully evaluated for psychometric properties. These questionnaires are more appropriate for dry eye assessment; however, they have different purposes and are available in different languages. Therefore, healthcare providers should consider the characteristics of each dry eye questionnaire when selecting the most appropriate for a specific scenario.

Initially, this review identified 24 dry eye questionnaires. Among them, 11 questionnaires (OSDI, IDEEL, DEQS, UNC DEMS, CDERQOL, NEI VFQ-25, DEQ, CLDEQ, OSD, and TERTC-DEQ) included questions on HRQL. Some of the questionnaires lacking items on HRQL were created to investigate DED morbidity and prevalence. However, it is preferable to include questions on HRQL for a more accurate assessment of a patient’s condition [20]. However, the impact of HRQL on DED assessment varies; for example, depending on the disease duration of DED [12]. Therefore, it is important to comprehensively assess both the impact on HRQL and clinical findings in DED treatment. Seven of the included questionnaires (OSDI, IDEEL, DEQS, UNC DEMS, CDERQOL, NEI VFQ−25, and OCI) were fully evaluated for psychometric properties in accordance with the PRO guidance established by the FDA [21]. Most of the included questionnaires were created before the FDA guidance was established in 2009; a few of them satisfy the stipulated requirements for psychometric properties, including the OSDI, NEI VFQ-25, and OCI, whose psychometric properties have been verified. Regarding the other questionnaires, the OSD presents good psychometric properties for one section—the OSD-QoL—which is the only section with assured reliability and validity. The validity and reliability of the DEQ, DEQ-5, CLDEQ, CLDEQ-5, OSD, TERTC-DEQ, McMonnies questionnaire, Schein questionnaire, Women’s Health Study questionnaire, SPEED, and SANDE have not been verified and future studies should further examine these questionnaires. In 2013, a review of dry eye questionnaires was conducted [24]; however, multiple new questionnaires, including the DEQS, UNC DEMS, and CDERQOL, have been developed since then. These three new questionnaires include questions on HRQL and were created in accordance with the PRO guidance. The development of these questionnaires could reflect the importance of determining the HRQL and PRO for dry eye evaluation.

The most common objective of the reviewed questionnaires (excluding the assessment of subjective symptoms) was HRQL evaluation (11 items), followed by disease severity (eight questionnaires: DEQ, CLDEQ, TERTC-DEQ, OCI, SANDE, DEQ-5, CLDEQ-8, SSI), prevalence rate (four questionnaires: Schein questionnaire, Women’s Health Study questionnaire, CANDEES, McCarty symptom questionnaire), and eye symptoms frequency (three questionnaires: OCI, SPEED, SANDE). Moreover, there are dry eye questionnaires (CLDEQ, CLDEQ-5) specifically designed to assess contact lens wearers. Therefore, it is important to select the appropriate questionnaire for each scenario.

The number of questions in each questionnaire ranged from 1 (UNC DEMS, SIDEQ) to 80 (OSD), with a median of 12 questions. Fifteen questionnaires had ≤20 questions. The inclusion of more translated questions could allow the collection of more detailed data regarding a patient’s status. However, there is an inevitable concomitant increase in the answering and scoring times. Moreover, a greater number of questions may increase recall bias [108,109]. The UNC DEMS and SIDEQ include one question each, whereas the SANDE questionnaire includes two questions. Moreover, the UNC DEMS and SANDE are correlated with the OSDI, which indicates that highly reliable information could be obtained from a small number of questions. The DEQ-5 and CLDEQ-8 are short versions of the DEQ and CLDEQ, respectively, with a reduced time burden compared to their long versions. It is important to select questionnaires with an appropriate number of questions for particular clinical settings, including time restriction, since the allotted time for answering and evaluating the questionnaire may vary.

The OSDI, DEQS, TERTC-DEQ, McMonnies questionnaire, DEQ-5, and CLDEQ-8 have designated DED cutoff values. Although subjective symptoms are required for DED diagnosis [12,13], questionnaires that lack cutoff values cannot provide a clear standard for determining whether the patient has subjective DED symptoms. Consequently, this judgment is left to the evaluator’s discretion. A questionnaire with a cutoff value is appropriate in cases requiring a DED diagnosis. The cutoff values for the OSDI are based on DED severity, whereas those for DEQ-5 are designated for the suspected Sjogren’s syndrome. These questionnaires are useful for a more detailed symptom assessment [30,43]. However, it is widely known that many patients with DED lack subjective symptoms; therefore, caution should be applied when diagnoses are made based on subjective symptoms [14,15,16,17]. In addition to evaluating subjective symptoms using a questionnaire, it is important to comprehensively evaluate the patient’s condition by combining the questionnaire results and clinical findings based on dry eye examinations, including TFBUT, ocular surface staining, the measurement of tear secretion using Schirmer’s test, and tear osmolarity [12,13]. The OSDI, DEQS, UNC DEMS, NEI VFQ−25, SPEED, DEQ-5, DEEP, and CLDEQ-8 are free to use [29,43,55,57,60,90,96,105]; however, the IDEEL must be purchased by the user [50]. The OSD-QoL is also not free for use, and its developers have mandated that all clinicians and researchers request permission prior to usage [74]. Other questionnaires have not directly indicated whether they require license fees in their initial publication [12,54,73,76,78,80,83,87,92,95,97,99,103].

This study has a limitation. This review was not intended to be a comprehensive and systematic review of the evidence regarding dry eye questionnaires. Rather, it was primarily designed to highlight the characteristics of dry eye questionnaires and the inclusion of questions regarding HRQL. Therefore, its focused and limited scope may not provide a comprehensive understanding of the functionality of various questionnaires for other uses.

This review identified six excellent dry eye questionnaires, namely the OSDI, IDEEL, NEI VFQ-25, CDERQOL, DEQS, and UNC DEMS. However, all the questionnaires have their limitations. The OSDI has only 12 questions, which renders it comparatively short with a low time burden; however, it is difficult to determine whether it allows a comprehensive evaluation of all dry eye symptoms and HRQL considerations. Contrastingly, the IDEEL has many questions (57 questions) with comprehensive coverage. However, it takes a long time to complete, which causes a large burden on both patients and medical institutions. The NEI VFQ-25 is a questionnaire unique to ophthalmology that applies to various diseases and conditions. However, its validity and reliability for patients with DED remain unclear. There is a need for future studies to determine whether the questionnaire is suitable for DED with respect to PROs. The DEQS, CDERQOL, and UNC DEMS are relatively new questionnaires that have been developed in accordance with the FDA guidance. However, they have several limitations, including the small sample sizes of the studies investigating their validity and reliability. Therefore, there is a need for further studies on these questionnaires. The CDERQOL and UNC are Chinese questionnaires, whereas the DEQS is a Japanese questionnaire. Therefore, these questionnaires should be validated in other languages in the future.

## 4. Conclusions

Preferably, dry eye questionnaires should include questions on HRQL and be evaluated for psychometric properties in accordance with the PRO guidance. Six of the twenty-four questionnaires identified in this review (OSDI, IDEEL, DEQS, UNC DEMS, CDERQOL, and NEI VFQ-25) satisfied both requirements. For dry eye assessment, it is essential to assess subjective symptoms and their effects on HRQL using questionnaires and clinical examination. However, there are differences among these questionnaires with respect to their purpose and number of questions for DED. Moreover, each questionnaire has its limitations, which emphasizes the need for medical practitioners to clearly understand the characteristics of each questionnaire before selecting the most appropriate one.

## Figures and Tables

**Table 1:** Classification of dry eye questionnaires according to patient-reported outcomes (PROs) and health-related quality of life (HRQL).

		HRQL	
		(+) *^1^	(−) *^2^
**PROs**	Sufficient *^3^	OSDI [29] IDEEL [48] DEQS [55] UNC DEMS [57] CDERQOL [54] NEI VFQ−25 [60]	OCI [78]
	Insufficient *^4^	DEQ [73] OSD [74] TERTC-DEQ [76]	McMonnies questionnaire [80] Schein questionnaire [83] Women’s Health Study questionnaire [87] SPEED [90] SANDE [92] DEQ-5 [43]
	No description *^5^	CLDEQ [73] 11-question dry eye syndrome questionnaire [12]	CANDEES [95] DEEP [96] McCarty symptom questionnaire [97] SSI [99] SIDEQ [103] CLDEQ-8 [105]

*1: Includes questions on HRQL; *2: Does not include questions on HRQL; *3: Validity and reliability are shown in accordance with the PRO guidance; *4: Insufficient description of psychometric properties or poor validity and reliability; *5: No description of psychometric properties. OSDI: Ocular Surface Disease Index, IDEEL: Impact of Dry Eye in Everyday Life, DEQS: Dry Eye-Related Quality-of-life Score, UNC DEMS: University of North Carolina Dry Eye Management Scale, CDERQOL: Chinese version of Dry Eye-Related Quality of Life, NEI VFQ-25: 25-Item National Eye Institute Visual Function Questionnaire, OCI: Ocular Comfort Index, DEQ: Dry Eye Questionnaire, OSD: Ocular Surface Disease, TERTC-DEQ: Texas Eye Research and Technology Center Dry Eye Questionnaire, SPEED: Standard Patient Evaluation of Eye Dryness questionnaire, SANDE: Symptom Assessment in Dry Eye, DEQ-5: Dry Eye Questionnaire-5, CLDEQ: Contact Lens Dry Eye Questionnaire, CANDEES: Canadian Dry Eye Epidemiology Study, DEEP: Dry Eye Screening questionnaire for Dry Eye Epidemiology Projects, SSI: Sicca Symptom Inventory, SIDEQ: Single Item Score Dry Eye Questionnaire, and CLDEQ-8: Contact Lens Dry Eye Questionnaire-8.

**Table 2:** Summary of the characteristics of the questionnaires.

Questionnaire	Aim	Development (Year)	Items	Scale	Psychometric Properties	Cutoff Value	Validated Language
OSDI [29]	Symptoms, HRQL, severity	1997	12	0–100 (for the total score and each subscale)	○	Mild 13–22; moderate 23–32; severe 33–100	English, Portuguese, [33], Spanish [34], Farsi [35], Bahasa [36], Chinese [37], Filipino [38], Japanese (J-OSDI) [39]
IDEEL [50]	Symptoms, HRQL, treatment satisfaction	2003	57	0–100 (for each module)	○	None	English
DEQS [55]	Symptoms, HRQL	2013	15	0–100	○	15	Japanese
UNC DEMS [57]	Symptoms, HRQL	2014	1	0–10	○	None	English
CDERQOL [54]	Symptoms, HRQL, treatment satisfaction	2017	45	5–225	○	None	Chinese
NEI VFQ−25 [60]	Visual function, HRQL	2001	25	0–100 (for each question)	○	None	>50 languages (original language is English) [63,64,65,66,67,68,69,70,71]
DEQ [73]	Symptoms, HRQL severity,	2001	21	N/A	△	None	English
OSD [74]	Symptoms, HRQL	2002	80	0–100 (for each section)	△	None	French, English [75]
TERTC-DEQ [76]	Symptoms, HRQL, identify patients with moderate dry eye, severity	2005	28	0–60	△	32.3	English
CLDEQ [73]	Symptoms with contact lens, HRQL,	2001	36	N/A	△	None	English
11-question dry eye syndrome questionnaire [12]	HRQL	2007	11	N/A	×	None	English
OCI [78]	Symptoms, discomfort, frequency, severity	2007	12	0–100	○	None	English, Chinese [79] (OCI-C)
McMonnies questionnaire [80]	Symptom, screening, risk factors	1986	12	0–45	△	14.5	English, Chinese [37]
Schein questionnaire [83]	Symptoms, prevalence in the elderly	1997	6	Not scored *	△	None	English
Women’s Health Study questionnaire [87]	Symptoms, prevalence in women	2003	3	Not scored *	△	None	English
SPEED [90]	Symptoms, frequency	2005	20	0–28	△	None	English
SANDE [92]	Symptoms, frequency, severity	2007	2	0–100	△	None	English
DEQ-5 [43]	Symptoms, severity	2009	5	0–22	△	>6 suspected dry eye; >12 suspected Sjogren’s syndrome	English, Spanish [94]
CANDEES [95]	Symptoms, prevalence	1997	13	Not scored *	×	None	English
DEEP [96]	Symptoms, screening	1998	19	0–114	×	None	English
McCarty symptom questionnaire [97]	Symptoms, prevalence	1998	6	0–18	×	None	English
SSI [99]	Symptoms, severity	2002	42/10	N/A	×	None	English, Portuguese [101]
SIDEQ [103]	Symptom, discomfort	2003	1	0–4	×	None	English
CLDEQ-8 [105]	Symptoms with soft contact lens	2009	8	0–37	△	12	English, Japanese [107] (J-CLDEQ-8)

○: Good validity and reliability according to the PRO guidance; △: Insufficient description of psychometric properties or poor validity and reliability; ×: No description of psychometric properties; N/A: Not applicable, score calculation method is not mentioned; Not scored *: There is no score for each question item. J-OSDI: Japanese version of the Ocular Surface Disease Index, OCI-C: Chinese version of the Ocular Comfort Index, J-CLDEQ-8: Japanese Contact Lens Dry Eye Questionnaire-8.

## Abbreviations

CANDEES	Canadian Dry Eye Epidemiology Study
CDERQOL	Chinese version of Dry Eye-Related Quality of Life
CLDEQ	Contact Lens Dry Eye Questionnaire
CLDEQ-8	Contact Lens Dry Eye Questionnaire-8
DED	Dry Eye Disease
DEEP	Dry Eye Screening questionnaire for Dry Eye Epidemiology Projects
DEQ	Dry Eye Questionnaire
DEQ-5	Dry Eye Questionnaire-5
DEQS	Dry Eye-Related Quality-of-life Score
FDA	Food and Drug Administration
HRQL	Health-Related Quality of Life
ICC	Intraclass Correlation Coefficient
IDEEL	Impact of Dry Eye in Everyday Life
J-CLDEQ-8	Japanese Contact Lens Dry Eye Questionnaire-8
J-OSDI	Japanese version of the Ocular Surface Disease Index
MCID	Minimal Clinically Important Difference
MGD	Meibomian Gland Dysfunction
N/A	Not Applicable
NEI VFQ-25	25-Item National Eye Institute Visual Function Questionnaire
OCI	Ocular Comfort Index
OCI-C	Chinese version of the Ocular Comfort Index
OSD	Ocular Surface Disease
OSD-QoL	OSD Quality of Life
OSDI	Ocular Surface Disease Index
PRO	Patient-Reported Outcome
QoL	Quality of Life
SANDE	Symptom Assessment in Dry Eye
SESoD	Subjective Evaluation of Symptom of Dryness
SIDEQ	Single Item Score Dry Eye Questionnaire
SPEED	Standard Patient Evaluation of Eye Dryness questionnaire
SSI	Sicca Symptom Inventory
TERTC-DEQ	Texas Eye Research and Technology left Dry Eye Questionnaire
TFBUT	Tear Film Break Up Time
UNC DEMS	University of North Carolina Dry Eye Management Scale
VAS	Visual Analog Scale
